# The rule of thirds: Determining the ideal areolar proportions

**DOI:** 10.1016/j.jpra.2019.11.001

**Published:** 2019-11-13

**Authors:** Matthew J. Laschuk, Linden K. Head, Grayson A. Roumeliotis, Lisa Xuan, Howard J. Silverman

**Affiliations:** a Division of Plastic and Reconstructive Surgery, Department of Surgery, Faculty of Medicine, University of Ottawa, The Ottawa Hospital – Civic Campus, 1053 Carling Avenue Box 213, K1Y 4E9 Ottawa, ON, Canada; b Oxford Craniofacial Unit, Oxford University, United Kingdom; c Faculty of Medicine, University of Ottawa, Canada

**Keywords:** Areola, Nipple-areolar complex, NAC, Breast surgery, Aesthetic surgery

## Abstract

**Background:**

Breast surgery often requires changing the diameter of the areola. Recommended areolar size is commonly based on population averages, or surgical judgement. An ideal areola size has not been previously been described. We hypothesized that the ideal areolar diameter would be proportional to two breast measurements not commonly altered during breast surgery: the nipple diameter and breast base width.

**Methods:**

‘The Sun’ newspaper (London, UK) publishes photographs of topless models which are selected based on the aesthetic appeal of their non-operated breasts. The publication's archive, from March 2014 to January 2017, was independently reviewed by three authors to identify photographs that presented a clear anterior view of the breast. The base width, nipple diameter and areolar diameter were measured independently by each reviewer. Measurements were pooled, and the mean was included for analysis. Ratios of the areolar diameter to the base width and the nipple diameter were calculated.

**Results:**

The photographs of 58 models were eligible for inclusion. The average areolar diameter to base width was 0.29 (SD = 0.05). The average nipple to areolar diameter was 0.29 (SD = 0.06).

**Conclusions:**

In aesthetically pleasing breasts, the areolar diameter is proportional to both the breast base width and nipple diameter. Breast base width is commonly measured preoperatively in aesthetic breast procedures, and is not typically modified. Breast base width can therefore be used to determine the ideal areolar size using the ratio of areola:base width ratio of 0.29 identified in this study.

## Introduction

Ideal breast aesthetics are quantified by several different metrics:^1^ Optimal breast volume, shape, nipple angulation,^2^^,^^3^ and breast parenchymal distribution^2^^,^^3^ have been defined. In surgical procedures in which the size of the nipple areolar complex will be altered, a final diameter of 35–45 mm is typically recommended.^4^ The typical ratio of the diameter of the nipple areolar complex to base width has also been described as 1:3.^5^ However, these reference values are based on normative data and describe population averages rather than aesthetically ideal breasts.^4^^,^^5^ The aesthetically ideal areolar diameter has not been defined.

Malluci and Branford^2^^,^^3^ applied a novel method to develop their widely accepted rule of vertical proportion of parenchymal distribution relative to the nipple meridian, and measured these variables in models who had been independently selected largely for the appearance of their non-operated breasts.^2^^,^^3^^,^^6^ We hypothesized that the aesthetically ideal diameter of the nipple areolar complex would be different from previous estimates based on population averages and would vary with breast base width.

## Methods

### Photograph selection

‘*The Sun’* newspaper (London, UK) regularly published photographs of topless models on *‘Page 3ʼ*. Models for ‘Page 3ʼ are selected based on the aesthetic appeal of their non-operated breasts.^2^^,^^3^^,^^6^ Because of the large international following and the independent popularity of ‘Page 3ʼ these models are considered to represent a cohort of women with ideal breast aesthetics.^2^^,^^3^

All available photographs from ‘The Sun’ newspaper website were reviewed for inclusion by three independent reviewers (ML, GR, LH). This publicly available archive includes all photos from March 2014 through January 2017.^6^ Photos were selected for inclusion based on predefined inclusion criteria. Only those photos that demonstrated a clear frontal view of at least one breast that provided adequate visualization of the breast base width, the areola, and the nipple were included.

### Data collection and analysis

The breast base width, nipple diameter and areolar diameter were independently measured by each reviewer using Adobe Photoshop CS6^Ⓡ^ (Adobe Systems, San Jose, California, United States).

**Figure 1:**
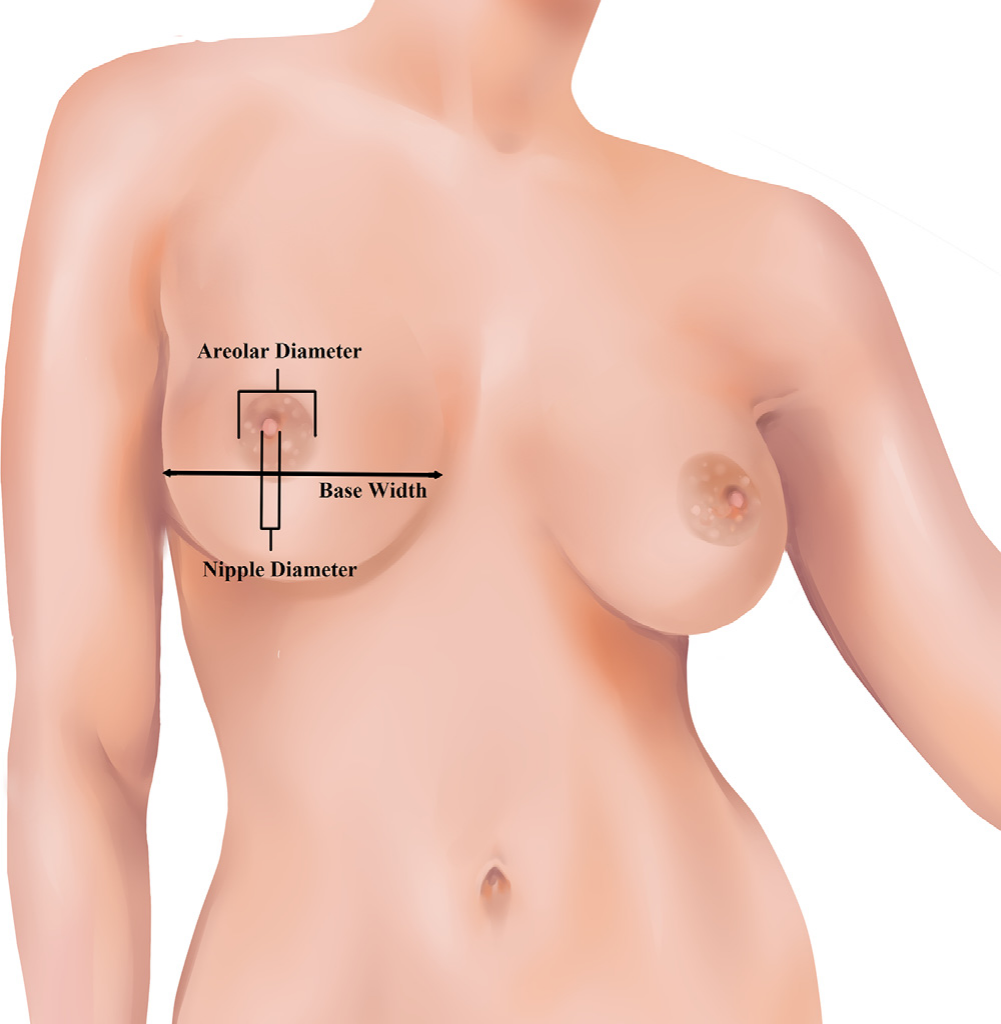
Breast measurements in the frontal view.

The base width was measured as the horizontal line at the level of the nipple drawn from the most medial footprint of the breast to the lateral arch of the breast mound (Figure 1). The areola was measured in its largest dimension in the horizontal and vertical axes to account for the vertical and horizontal asymmetry seen in the areolar diameter. The mean value of these measurements was used to represent the true areolar diameter. Similarly, the diameter of the nipple was measured in both the vertical and horizontal planes and the mean diameter used as the true nipple diameter. Breast base width was measured as the maximum width of the breast footprint on the chest wall. Ratios of each model's areolar diameter to base width, and areolar diameter to the nipple diameter, were calculated using Microsoft Excel (Redmond, Washington, United States). These ratios were pooled to calculate the mean.

## Results

A total of 58 photographs depicting 58 models met our criteria and were selected for inclusion. Agreement between reviewers was 100%. The mean areolar diameter to base width ratio was 0.29 with a standard deviation of 0.05. The mean nipple diameter to areolar diameter ratio was found to be 0.29 with a standard deviation of 0.06.

## Discussion

In a sample of 58 models selected largely for aesthetically pleasing breasts, the areola diameter to base width ratio was 0.29 and the nipple diameter to areolar diameter ratio was 0.29. Similar ratios have been described in a random sample of women.^5^ However, by measuring these variables in a population of women selected for the appearance of their breasts the outcome more likely represents an aesthetic ideal rather than a population average. While measurement based on non-standardized photographs is suboptimal, this is an accepted technique that has been previously applied in this context.^2^^,^^3^

These ratios are clinically relevant to aesthetic and reconstructive breast surgeons because while areolar size is routinely changed during breast surgery, the nipple diameter and base width of the breast are rarely altered: The base width of the breast decreases by only 0.0 cm ± 0.4 cm per 100 g resection weight^7^ following vertical reduction mammoplasty. These ratios can therefore be used to accurately plan the desired areola size pre- or intraoperatively based on breast base width. To simplify this ratio, we feel a ‘rule of thirds’ can be applied to breast aesthetics, with the areola representing just under one third the base width and the nipple representing approximately one third of the areolar diameter.

## Conclusions

The aesthetically ideal areolar diameter is related to both the base width of the breast and the nipple diameter. The ‘rule of thirds’ ratios should be considered when surgically altering the size of the areolar complex and represents a useful tool to aid the reconstructive or cosmetic breast surgeon to achieve the aesthetic goals of their patients.

## Funding

None.

## Commercial associations or financial disclosures

None.

## Products referenced

None.

## CRediT authorship contribution statement

**Matthew J. Laschuk:** Conceptualization, Data curation, Formal analysis, Methodology, Validation, Writing - original draft, Writing - review & editing. **Linden K. Head:** Conceptualization, Data curation, Formal analysis, Methodology, Validation, Writing - original draft, Writing - review & editing. **Grayson A. Roumeliotis:** Conceptualization, Data curation, Formal analysis, Methodology, Validation, Writing - original draft, Writing - review & editing. **Lisa Xuan:** Conceptualization, Formal analysis, Methodology, Validation, Writing - original draft, Writing - review & editing. **Howard J. Silverman:** Conceptualization, Formal analysis, Methodology, Validation, Writing - original draft, Writing - review & editing.

## Declaration of Competing Interest

There are no conflicts of interest to disclose by any of the involved authors. No external sources of funding, support, or benefits were received by any of the authors.

## References

[1] Kim M.S., Sbalchiero J.C., Reece G.P. (2008). Assessment of breast aesthetics. Plast Reconstr Surg.

[2] Mallucci P., Branford O.A. (2014). Population analysis of the perfect breast: a morphometric analysis. Plast Reconstr Surg.

[3] Mallucci P., Branford O.A. (2012). Concepts in aesthetic breast dimensions: analysis of the ideal breast. J Plast Reconstr Aesthetic Surg.

[4] Mathes S.J. (2006). UC. Reconstruction of the nipple-areola complex. Plastic surgery VI.

[5] Hauben D.J., Adler N., Silfen R., Regev D. (2003). Breast-areola-nipple proportion. Ann Plast Surg.

[6] The Sun. http://www.page3.com. Published 2018. Accessed November 19, 2017.

[7] Smithson M.G., Collawn S.S., Mousa M.S., Bramel C.M. (2017). A formula for planning and predicting postoperative mammoplasty results. Ann Plast Surg.

